# Investigation of Microcystin Congener–Dependent Uptake into Primary Murine Neurons

**DOI:** 10.1289/ehp.0901289

**Published:** 2010-05-14

**Authors:** Daniel Feurstein, Julia Kleinteich, Alexandra H. Heussner, Kerstin Stemmer, Daniel R. Dietrich

**Affiliations:** Human and Environmental Toxicology, University of Konstanz, Konstanz, Germany

**Keywords:** cyanobacteria, microcystin congeners, neurotoxicity, organic anion-transporting polypeptides

## Abstract

**Background:**

Contamination of natural waters by toxic cyanobacteria is a growing problem worldwide, resulting in serious water pollution and human health hazards. Microcystins (MCs) represent a group of > 80 cyclic heptapeptides, mediating cytotoxicity via specific protein phosphatase (PP) inhibition at equimolar concentrations (comparable toxicodynamics). Because of the structure and size of MCs, active uptake into cells occurs via organic anion-transporting polypeptides (OATP/Oatp), as confirmed for liver-specific human *OATP1B1* and *OATP1B3*, mouse *Oatp1b2* (m*Oatp1b2*), skate *Oatp1d1*, and the more widely distributed *OATP1A2* expressed, for example, at the blood–brain barrier. Tissue-specific and cell-type–specific expression of OATP/Oatp transporters and specific transport of MC congeners (toxicokinetics) therefore appear prerequisite for the reported toxic effects in humans and other species upon MC exposure. Beyond hepatotoxicity induced by the MC-LR congener, the effects of other MC congeners, especially neuronal uptake and toxicity, are unknown.

**Objectives:**

In this study we examined the expression of mOatps and the uptake of congeners MC-LR, MC-LW, and MC-LF in primary murine neurons.

**Methods:**

Intracellular MC accumulation was indicated indirectly via uptake inhibition experiments and directly confirmed by Western blot analysis and a PP inhibition assay. Neuronal mOatp expression was verified at the mRNA and protein level.

**Results:**

MCs can cross neuronal cell membranes, with a subsequent decrease of PP activity. Of 15 mOatps, 12 were expressed at the mRNA level, but we found detectable protein levels for only two: m*Oatp1a5* (*Slco1a5*) and the known MC-LR transporter m*Oatp1b2* (*Slco1b2*).

**Conclusions:**

These data suggest mOatp-mediated uptake of MC congeners into neurons, thus corroborating earlier assumptions of the neurotoxic potential of MCs.

Cyanobacteria are abundant in marine, brackish, and fresh waters. During the past decades, cyanobacterial poisonings of aquatic and terrestrial animals as well as humans (Kuiper-Goodman et al. 1999) have been reported worldwide, especially in conjunction with cyanobacterial blooms in water reservoirs, rivers, lakes, and ponds used for drinking water or recreational purposes or as water resources for livestock. Current research suggests that climate change, specifically global warming, will promote the incidence and severity of cyanobacterial mass occurrences (Carmichael 2008; Paerl and Huisman 2008; Paul 2008), thereby increasing the potential threat posed by cyanobacterial toxins to human health and livestock (World Health Organization 1999).

Microcystin (MC), the most common freshwater cyanotoxin, represents a group of cyclic heptapeptides encompassing > 80 structural variants (Meriluoto and Spoof 2008), with molecular weights ranging between 900 and 1,100 Da (Zurawell et al. 2005). Their inherent capability for inhibiting serine/threonine-specific protein phosphatases (PPs; e.g., PP1, PP2A, PP4, PP5) via covalent binding to the PP catalytic subunit (Hastie et al. 2005) results, once taken up into the cell, in a disruption of cellular phosphorylation/dephosphorylation homeostasis. This in turn leads to several downstream responses, such as disintegration of cytoskeletal structure, inhibition of gluconeogenesis, and enhanced glycolysis (Batista et al. 2003; Falconer and Yeung 1992), frequently with later onset of apoptosis and necrosis (Fladmark et al. 1999; Komatsu et al. 2007). However, to exert the MC-specific PP inhibition, sufficient concentrations of MC must enter the cell. Cellular uptake of MCs has been demonstrated to occur exclusively via an active transport, whereas passive transmembrane diffusion can be excluded (Eriksson et al. 1990; Fischer et al. 2005; Komatsu et al. 2007). Consequently, pathological changes following MC intoxications are restricted to organs, tissues, and cells capable of actively transporting MC from the blood into the cell. Indeed, active transmembrane transport of MCs is mediated by specific organic anion-transporting polypeptides (human OATP/rodent Oatp). Approximately 80 OATPs/Oatps have been identified from 13 different species. In the human, mouse, and rat, 36 different OATPs/Oatps have been found (Hagenbuch 2007; Hagenbuch and Meier 2004); these can be expressed either ubiquitously (e.g., OATP1A2) or with tissue/organ-specific expression (e.g., *OATP1B1* and *1B3* in the liver) (Hagenbuch and Meier 2004). However, not all OATPs/Oatps are capable of transporting MCs (Fischer et al. 2005), and different OATPs/Oatps appear to have largely differing affinities and capacities for MC congeners (Feurstein et al. 2009; Monks et al. 2007). Thus, these differing affinities and capacities highlight the fact that OATPs/Oatps capable of transporting MCs need to be functionally expressed in a tissue/organ or cell type such that MCs can exert a cytotoxic effect. Indeed, this has been demonstrated convincingly with *Oatp1b2* knockout mice, which were resistant to the overt hepatotoxicity of the MC congener MC-LR observed in corresponding wild-type mice (Lu et al. 2008). In consequence, the often-quoted hepatotoxicity and nephrotoxicity of MCs are the result of a hepatic first-pass and subsequent renal elimination effect in organs having a high level of functionally expressed OATPs/Oatps capable of MC transport. More recently, several OATPs/Oatps were described in the blood–brain barrier (BBB), in the blood–cerebrospinal fluid barrier (BCSFB), in human gliomas, and in glia cells (Bronger et al. 2005; Huber et al. 2007; Westholm et al. 2009). Therefore, it may be assumed that MCs are able to enter the brain and to exert neurotoxic effects. Indeed, 116 (89%) of 131 patients of a hemodialysis unit in Caruaru, Brazil, accidentally exposed to MC congeners via dialysis water (specifically MC-LR, MC-YR, and MC-AR) (Carmichael et al. 2001; Pouria et al. 1998) presented with acute symptoms of neurotoxicity (e.g., deafness, tinnitus, reversible blindness). Subsequently, 100 patients developed liver failure, of which 76 died (Carmichael et al. 2001; Pouria et al. 1998). Furthermore, a reduction in brain size was reported in progeny of Swiss Albino mice exposed to cyanobacterial bloom extract containing MCs (Falconer et al. 1988), thus suggesting that MCs have an effect on the brain. Whether the observed neurological effects in the Caruaru patients stem from an effect of MCs on the endothelium of the BBB with subsequent *in situ* ischemia and inflammatory reactions, or a direct uptake of MCs via OATPs of the BBB endothelium (Cecchelli et al. 2007) and OATPs of astrocytes, microglia, and/or neurons, remains to be resolved. Recently, acute MC congener–dependent cytotoxicity as well as the presence of murine Oatps (mOatps) was demonstrated in primary murine whole-brain cells (Feurstein et al. 2009); however, that study did not differentiate between the various cell types affected, such as astrocytes, microglia, or neurons. Thus, although the literature strongly suggests the presence of OATPs/Oatps capable of MC transport in the BBB, the expression of OATPs/Oatps in neurons and MC congener neurotoxicity still remain elusive. In view of the scarcity of human primary neurons, we used mouse primary neurons to determine the identity of mOatps expressed, to confirm mOatp-mediated MC congener-specific uptake, and to determine MC congener–specific inhibition of neuronal PPs.

## Materials and Methods

### Materials

We obtained [^3^H]taurocholate (TC; 170.2 GBq/mmol) and [^3^H]estrone sulfate ammonium salt (ES; 2,120 GBq/mmol) from PerkinElmer Life and Analytical Sciences (Boston, MA, USA). MC congeners were from Alexis Biochemicals (Lausen, Switzerland), and reverse-transcriptase polymerase chain reaction (RT-PCR) chemicals were from Fermentas (St. Leon-Roth, Germany). All other chemicals and antibodies, unless otherwise stated, were from Sigma-Aldrich (Taufkirchen, Germany). Cell culture media and reagents were obtained from PAA Laboratories (Cölbe, Germany).

### Isolation and cultivation of primary neurons

Specific pathogen-free Balb/c mice were obtained from the Jackson Laboratory (Bar Harbor, ME, USA) and held at the animal facility at the University of Konstanz under standard conditions. Sacrifice and organ removal were carried out humanely and with regard for alleviation of suffering in accordance with the German Animal Protection Law, approved by the Regierungspräsidium in Freiburg, Germany (registry no. T-07 05). Primary murine cerebellar granule neurons (Skaper et al. 1996) were isolated and cultured as described previously (Volbracht et al. 1999). Briefly, cerebelli from 6- to 7-day-old pups were removed, cells were separated, and 2.0 × 10^6^ cells/mL were seeded in poly-l-lysine (50 mg/L)–coated plates using basal medium Eagle (BME), supplemented with 10% fetal calf serum, 20 mM potassium chloride, and 1% penicillin-streptomycin and cultured at 37°C in 5% carbon dioxide. Medium containing 10 μM cytosine arabinoside to inhibit growth of glial cells was added after the initial 24 hr, and cells were grown for 3 days without further medium exchange.

### Western blot (WB) analysis for MC-LR detection

Neurons were exposed to varying MC-LR concentrations for 48 hr, and 30 μg of each sample was prepared for WB analysis as previously described (Feurstein et al. 2009). We used anti-MC-LR [polyclonal rabbit anti-MCLR#2; 1:500 (Mikhailov et al. 2001)] as the primary antibody, anti-glyceraldehyde-3-phosphate dehydrogenase (anti-GAPDH; monoclonal mouse anti-GAPDH, 1:30,000) as a housekeeping control protein, and horseradish peroxidase–conjugated secondary antibodies [rabbit anti-mouse (1:80,000) and goat anti-rabbit (1:160,000)]

### PP activity after exposure to single MC congeners

Cells were exposed to varying concentrations of single MC congeners for 48 hr and solubilized in 50 μL enzyme solution buffer [0.08 M Tris-HCl (pH 7.0), 0.19 mM ethylene glycol tetraacetic acid, 20 mM dithiothreitol (DTT; in 0.01 M sodium acetate, pH 5.2), 10 mM manganese chloride (MnCl_2_)]. Protein concentrations were determined using the method of Bradford (1976). Subsequently, 20 μL of each sample (containing 10 μg total protein) was transferred to a 96-well plate, and an equal volume of water was added. To determine PP activity, 200 μL freshly prepared and prewarmed (37°C) substrate solution containing reaction buffer [0.25 M Tris-HCl (pH 8.1), 10 mM MnCl_2_, 0.2 M magnesium chloride, 5 mg/mL bovine serum albumin (BSA)], 60 mM *p*-nitrophenyl phosphate (pNPP; Acros Organics, Morris Plains, NJ, USA), and 20 mM DTT were added and immediately measured at 405 nm (TECAN infinite M200; TECAN, Craislheim, Germany) to determine the 0-time value. After incubation for 90 min at 37°C, we calculated total PP activity of MC-exposed neurons by determining the percent loss of pNPP substrate turnover from untreated cells (100% PP activity). Individual samples were analyzed in triplicate (*n* = 3), each with technical duplicates.

### Determination of mOatp mRNA expression in primary neurons

Total RNA was isolated using the RNeasy Mini Kit (Qiagen, Hilden, Germany) according to manufacturer’s instructions. RT-PCR was carried out in a final volume of 20 μL containing 10.5 μL total RNA (0.5 μg) diluted in RNase-free water, 1 μL random hexamer primer (50 μM), and 1 μL oligo(dT) (50 μM). The mixture was incubated at 70°C for 10 min to promote primer annealing. Subsequently, 5× reaction buffer (4 μL), 1 μL deoxynucleoside-triphosphate mix (10 mM and 2 μL DTT (0.1 M) were added and incubated at 42°C for 2 min. Finally, cDNA synthesis was carried out using 0.5 μL SuperScript II (100 U; Invitrogen, Karlsruhe, Germany) for 50 min at 42°C, followed by an inactivation for 15 min at 70°C. PCR was performed in 20 μL reaction mixtures according to Mühlhardt (2002), with minor modifications. Specific primers for mOatps [see Supplemental Material, Table 1 (doi:10.1289/ehp.0901289)] were designed using Primer3 software, version 0.4.0 (Rozen and Skaletzky 2000). mOatp amplification was carried out by mixing 1 μL cDNA product and using the following cycling conditions for all reactions: 4.5 min at 95°C, 35 cycles of 30 sec at 95°C, 1 min at 59°C, 1 min at 72°C, 1 min at 95°C, and 1 min at 55°C. Finally, each PCR product was separated by gel electrophoresis on a 3% agarose gel buffered in 10.8 g/L Tris-base, 5.5 g/L boric acid, and 4 mL (vol/vol) 0.5 M EDTA, pH 8.0. We used DNA ladder (GeneRuler 100bp; Fermentas, St. Leon-Rot, Germany) to determine the PCR product size. Gels were stained by ethidium bromide, visualized, and then scanned for documentation.

### WB analysis for mOatp detection

Primary mouse neurons were homogenized, subjected to mOatp-WB analysis, and visualized using chemiluminescence, as previously described (Feurstein et al. 2009). Homogenates of murine whole brains and livers served as positive controls for mOATP expression. Briefly, separated protein samples (15 μg) were transferred to a nitrocellulose membrane. Polyclonal goat antibodies against OATP-D (mOatp3a1), OATP-3 (mOatp1a5), OATP-E (mOatp4a1), OATP-F (mOatp1c1), OATP-B (mOatp2b1) (1:100; all from Santa Cruz Biotechnology, Santa Cruz, CA, USA), and mOatp1b2 (1:1,000; kindly provided by C.D. Klaassen, University of Kansas Medical Center, Kansas City, KS, USA), and monoclonal mouse anti-β-actin (1:1,000) were used as primary antibodies and incubated for 16 hr at 4°C. The horseradish peroxidase–conjugated secondary antibodies—goat anti-rabbit (1:160,000), goat anti-mouse (1:80,000), and rabbit anti-goat (1:80,000)—were incubated for 1 hr at room temperature (RT). Immunoreactive bands were detected by enhanced chemiluminescence (ECL Plus; GE Healthcare, Munich, Germany).

### Immunocytochemistry for mOatp1b2 detection

Cells were fixed in 4% paraformaldehyde for 15 min at RT, permeabilized (0.2% Triton X-100), and subsequently incubated with blocking buffer [phosphate-buffered saline (PBS) containing 1% BSA] for 1 hr at RT. Polyclonal rabbit anti-mOatp1b2 (1:800) was applied, and slides were incubated for 16 hr at 4°C. Secondary fluorochrome-conjugated goat anti-rabbit Alexa Fluor 647 antibody (Invitrogen) was applied in a 1:1,000 dilution and incubated for 1 hr at RT. Nuclei were counterstained with 2.5 μM Hoechst 33342 (Invitrogen) for 10 min at RT. Finally, slides were visualized using an Axiovert 200M microscope (Zeiss, Göttingen, Germany).

### TC and ES uptake studies

The transport capacity and potential functionality of neuronal mOatps were assessed using [^3^H]TC and [^3^H]ES uptake. [^3^H]TC and [^3^H]ES are well-characterized substrates for and thus transported by a variety of OATPs/Oatps (Konig et al. 2006; van Montfoort et al. 2003). Uptake experiments were initiated by addition of 500 μL BME medium prewarmed to 37°C and containing varying concentrations of either [^3^H]TC (3.7 kBq/mL) or [^3^H]ES (3.7 kBq/mL) and unlabeled TC (7, 40, 60, 80, and 100 μM) and ES (7, 10, 20, 30, 40, and 50 μM), respectively. After 30 min exposure at 37°C, uptake was stopped by removing the medium and performing a subsequent washing step using 3× 1.5 mL ice-cold PBS per well. Neurons were solubilized with 500 μL 0.2 normal sodium hydroxide per well (15 min), and the solution was neutralized with 500 μL 0.2 normal HCl. Solubilized neurons were mixed with 10 mL scintillation cocktail (Ready Safe; Beckman Coulter, Krefeld, Germany), and radioactivity was determined in a liquid scintillation counter (LS 6500; Beckman Coulter). To normalize radioactivity per milligram of protein, we determined protein concentration according to the method of Bradford (1976). Individual samples were analyzed in triplicate (*n* = 3), each with technical duplicates.

### MC congener–dependent inhibition of TC/ES uptake

Uptake inhibition experiments were carried out as described above, but using 500 μL BME medium containing either 7 μM of a mixture of [^3^H]TC (3.7 kBq/mL) and TC or 7 μM of a mixture of [^3^H]ES (3.7 kBq/mL) and ES in the absence or presence of single MC congeners (7 μM). To normalize radioactivity per milligram of protein, we determined protein concentration as described above. Individual samples were analyzed in triplicate (*n* = 3), each with technical duplicates.

### Data analysis

We performed statistical analysis by one-way analysis of variance with Dunnett’s posttest using GraphPad Prism 4.03 (GraphPad Software Inc., La Jolla, CA, USA). The level of statistical significance was set at *p* < 0.05 or *p* < 0.01.

## Results

### Confirmation of intracellular MC-LR

**Figure 1:**
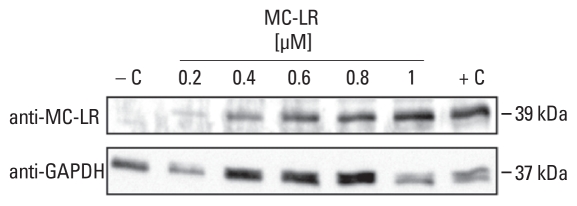
Intracellular- and concentration-dependent detection of MC-LR by WB analysis of neurons exposed to MC-LR for 48 hr; 30 μg total protein per lane. Abbreviations: −C, untreated neurons; +C, 1 μM MC-LR was added to a neuronal homogenate.

We confirmed MC-LR uptake into murine neurons by MC-LR WB analysis (Figure 1), which suggests cellular uptake of MC-LR. The single MC-LR–positive band, with an approximate molecular weight of 39 kDa, corresponded to the expected size of the PP1 (37.5 kDa) and PP2A (36 kDa) catalytic subunits with covalently bound MC-LR (1 kDa).

### Intracellular PP inhibition by MCs

**Figure 2:**
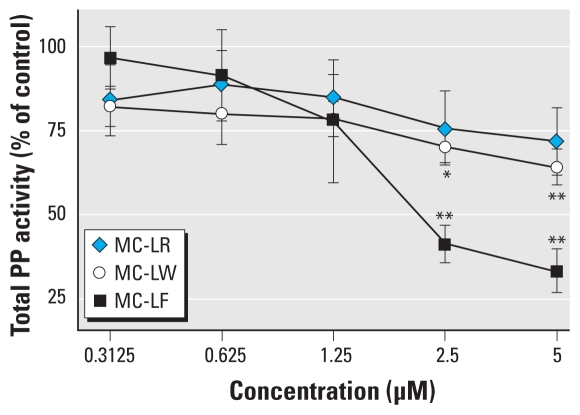
Total PP activity of primary neurons after 48 hr exposure to MC-LR, MC-LW, or MC-LF. Individual samples were analyzed in triplicate (*n* = 3), each with technical duplicates; data are mean ± SEM expressed as percent of control (untreated cells; 100% PP activity). *p < 0.05. **p < 0.01.

We directly confirmed MC congener transport into neurons using a colorimetric PP inhibition assay. At low MC concentrations (0.31–1.25 μM), the inhibitory effect of all MC congeners was comparable and resulted in a 20% reduction of PP activity compared with control (Figure 2). At 2.5 μM MC-LR, MC-LW, or MC-LF, total PP activity was reduced by 25%, 30%, and 60%, respectively, whereas 5 μM MC-LF reduced total PP activity by 65%.

### Determination of mOatp expression in primary murine neurons

**Figure 3:**
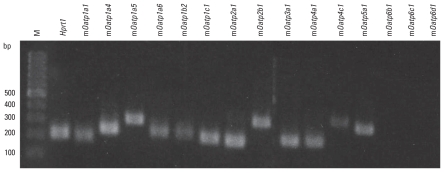
RT-PCR of *mOatp* mRNA expression in primary neurons. Abbreviations: *Hprt1*, housekeeping gene; M, DNA ladder in base pairs (bp).

We detected specific mRNA expression for 12 of 15 mOatps, as demonstrated by individual single positive bands (Figure 3) corresponding to the expected size of the amplified products (m*Oatp1a1*, *1a4*, *1a5*, *1a6*, *1b2*, *1c1*, *2a1*, *2b1*, *3a1*, *4a1*, *4c1*, and *5a1*). No mRNA expression was found for the three members of the m*Oatp6* family (m*Oatp6b1*, *6c1*, and *6d1*). Water and RT- negative controls indicated no contamination/bands (data not shown).

**Figure 4:**
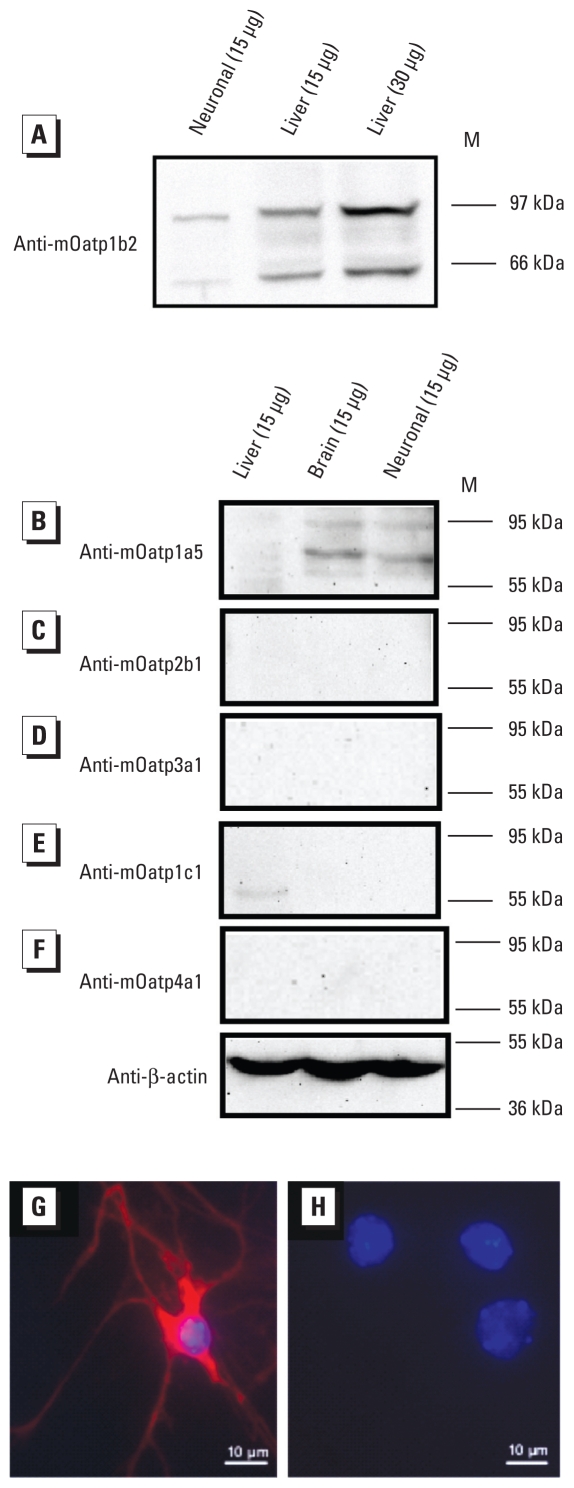
WB analysis in neuronal membrane fractions (*A–F*) and immunodetection of mOatp1b2 in cultured neurons (*G,H*). M, protein marker. (*A–F*) WB analysis of mOatp1b2 (*A*), mOatp1a5 (*B*), mOatp2b1 (*C*), mOatp3a1 (*D*), mOatp1c1 (*E*), and mOatp4a1 (*F*). Liver and brain membrane fractions were used as positive controls, and β-actin was a loading control. (*G,H*) Immunodetection of mOatp1b2 in cultured neurons treated with (*G*) Hoechst (blue), rabbit anti-mOatp1b2, and fluorochrome-conjugated secondary antibody (red) or (*H*) Hoechst and fluorochrome-conjugated secondary antibody only (negative control).

We analyzed mOatp expression at the protein level by WB using crude membrane fractions of primary murine neurons, as well as mouse liver and brain homogenates. The expression of mOatp1b2 and mOatp1a5 was confirmed in neurons (Figure 4A,B) with immunopositive bands at approximately 80 and 60 kDa and 85 and 65 kDa, respectively. Expression of mOatp2b1 (Figure 4C), mOatp3a1 (Figure 4D), mOatp1c1 (Figure 4E), and mOatp4a1 (Figure 4F) was either absent or less than the limit of detection for the crude neuron membrane extract we used.

We also assessed the expression and localization of mOatp1b2 in neurons via immunocytochemistry, which revealed immunopositive staining in membranes of the perikaryon as well as in neurites of cultured cells (Figure 4G). No unspecific binding of the fluorochrome-conjugated secondary antibody to neurons (negative control) was found (Figure 4H).

### Inhibition of TC/ES uptake by MC congeners

**Figure 5:**
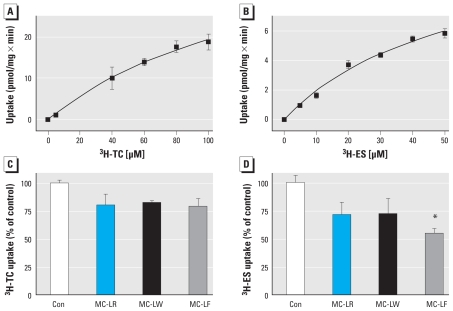
Uptake of [^3^H]TC (*A*) and [^3^H]ES (*B*) in neurons and the inhibitory effect of MC-LR, MC-LW, and MC-LF on uptake of [^3^H]TC (*C*) and [^3^H]ES (*D*). (*A* and *B*) Neurons were exposed to varying concentrations of labeled/unlabled TC and ES. (*C* and *D*) Cells were exposed to [^3^H]TC plus TC (7 μM; *C*) or [^3^H]ES plus ES (7 μM; *D*) in the absence (control; Con) or presence of a single MC congener (7 μM). The inhibitory effect was calculated by determining the percent loss of co-incubated versus TC- or ES-exposed neurons (100% uptake). Individual samples were analyzed in triplicate (*n* = 3), each with technical duplicates; data are mean ± SEM. *p < 0.05.

No typical substrate uptake curve resulted using either of the two Oatp/OATP substrates, TC and ES (Figure 5A,B). Moreover, contrary to expectations, even at the highest TC and ES concentrations (189 and 567 μM, respectively), no saturation of mOatps with substrate could be demonstrated (data not shown); this suggests unspecific binding of both TC and ES to neuronal cells (Figure 5A,B), effectively preventing detection of specific saturable uptake if present. Independent of the latter observations, TC and ES uptake in neurons appeared to be inhibited by MC-LR, MC-LW, and MC-LF. Although statistically not significant, TC uptake tended to be inhibited, irrespective of the MC congener used (Figure 5C). In contrast, whereas inhibition of ES uptake by MC-LR or MC-LW was similarly lower, as observed for TC, a statistically significant inhibition (45% of control) of ES uptake occurred with MC-LF (Figure 5D).

## Discussion

Eriksson et al. (1987) demonstrated that a cyclic peptide toxin isolated from the cyanobacterium *Microcystis aeruginosa* presented with a cell-type–specific cytotoxicity; they later showed that a multispecific bile acid transport system is involved in the uptake of MC-LR into primary rat hepatocytes (Eriksson et al. 1990). More recently, it was conclusively demonstrated that OATPs/Oatps are responsible for transporting MC-LR (Fischer et al. 2005, 2010; Komatsu et al. 2007; Lu et al. 2008; Meier-Abt et al. 2007). However, not all OATPs/Oatps are capable of transporting MC-LR (Fischer et al. 2005). Moreover, OATPs/Oatps decisively differ in their ability to transport the structurally variant MC congeners (Feurstein et al. 2009; Fischer et al. 2010; Monks et al. 2007). Thus, the potential neurotoxicity of individual MC congeners largely depends on the functional expression of OATPs/Oatps at the BBB/blood–cerebrospinal fluid barrier (Bronger et al. 2005; Huber et al. 2007; Westholm et al. 2009) and in the neuronal cell membrane. Another prerequisite is that OATPs/Oatps are capable of transporting MC congeners across these barriers.

Using the mouse as a human surrogate, we demonstrated the mRNA expression of 12 mOatps in primary murine neurons (Figure 3). However, we detected only two (m*Oatp1b2* and m*Oatp1a5*) of six tested mOatps in neuronal membranes at the protein level (Figure 4A,B). Indeed, WB analysis of mOatp1b2 and mOatp1a5 showed two positive bands, at approximately 80 and 60 kDa and 85 and 65 kDa, respectively, most likely representing the double bands of mOatps described in the literature as glycosilated/deglycosilated variants of the corresponding transporter (Hagenbuch and Meier 2004; Komatsu et al. 2007; Zaher et al. 2008). We assume that the absence of detectable protein expression in WB analyses of the other mOatps expressed at the mRNA level results either from expression levels that are too low to allow detection with the amount of crude neuron membrane extract used or from the complete absence of functional expression in neuronal membranes. Beyond the mOatps analyzed, the limited number of additional commercially available mOatp antibodies prevented detection of other mOatps possibly expressed in neuronal membranes.

Despite the proven expression of m*Oatp1b2* and m*Oatp1a5* in neuronal cells, and contrary to expectations and previous experience in transfected and primary cells and oocytes (Fischer et al. 2005, 2010), we found no saturable transport with the Oatp/OATP substrates TC and ES (Figure 5A,B). Although at first glance this seems contradictory, the latter observations may stem from experimental problems, for example, unspecific binding of TC and ES to neuronal membranes that prevented detection of saturable transport of mOatps expressed at very low levels (Figure 4). Indeed, evidence that mOatp- mediated transport of TC, ES, and MC congeners occurred stems from three separate lines of experimental data.

First, we detected covalently bound MC-LR by WB analysis of mouse neuron homogenate preparations after exposure to MC-LR (Figure 1). The bands detected corresponded to the molecular weights of the catalytic subunits of PP1 and PP2A with covalently bound MC-LR and were comparable to those for mice and murine whole brain cells reported previously by Lu et al. (2008) and Feurstein et al. (2009). Using MC-LR–treated wild-type and MC-LR–treated Oatp1b2^−/−^ mice, Lu et al. (2008) demonstrated that m*Oatp1b2* is a critical carrier of MC-LR in liver, but they also highlighted the fact that other mOatps can contribute to the hepatic uptake of MC-LR.

Second, variant MC transport by mOatps was suggested in the mouse neuron uptake inhibition experiments when we used the well-characterized OATP/Oatp substrates TC and ES. Because all of the OATPs/Oatps known to transport MC-LR are also established transporters of TC and ES (Konig et al. 2006; van Montfoort et al. 2003), it is not surprising that coincubations of TC or ES with equimolar concentrations of MC-LR, MC-LW, and MC-LF appeared to reduce uptake of TC and ES (Figure 5C,D). Contrary to expectations from previous experiments using primary human hepatocytes and OATP-transfected HEK293 cells (Fischer et al. 2010), the overall reduction in substrate uptake mediated by all MC congeners appeared to be at best marginal for TC, whereas we observed an effect with MC-LF for the reduction of ES uptake. The latter corroborates earlier findings by Fischer et al. (2010) that MC-LF is taken up more rapidly or efficiently by *OATP1B* family members than are other MC congeners.

Third, MC-LR, MC-LW, and MC-LF have been reported to have comparable toxicodynamic properties (PP inhibition) (Monks et al. 2007). In the present study, MC-LF induced the greatest reduction in PP activity after exposure of neuronal cells to equimolar concentrations of the three MC congeners (Figure 2), which suggests that MC-LF was transported more efficiently into the cell (toxicokinetics) and thus reached PP-inhibitive intracellular concentrations more quickly than did MC-LR and MC-LW. As noted above, more efficient transport of MC-LF was previously reported in OATP1B1- and OATP1B3-transfected HEK293 cells and primary human hepatocytes (Fischer et al. 2010), OATP-transfected HeLa cells (Monks et al. 2007), and murine whole-brain cells containing a mixture of neuronal cells, astrocytes, and microglia (Feurstein et al. 2009).

However, despite the evidence presented above suggesting neuronal uptake of MCs, it has yet to be elucidated whether oral MC exposure leads to neurotoxicity in mice *in vivo*. The relevance for the human situation is that, irrespective of the fact that our experiments were carried out in mouse primary neuronal cells and that mice are mere surrogates for humans, all experiments performed in this study employed relevant MC concentrations compared with those detected in the serum of human patients in Caruaru after exposure to MCs through dialysis water.

Moreover, the observations in primary murine neuronal cells presented here correspond well with earlier findings of OATP-mediated MC transport and cytotoxocity in primary human liver cells and *OATP1B1-* and *OATP1B3*-transfected HEK293 cells (Fischer et al. 2010). The latter lines of evidence thus strongly support the assumption at the outset that MC-induced neurotoxicity may have been involved in the neurological dysfunctions observed in Caruaru patients.

## Conclusion

All together, the data presented here strongly suggest that mOatps, albeit not exclusively mOatp1b2, are involved in the neuronal uptake of MCs and that the uptake and subsequent neurotoxicity are MC congener dependent. Provided these observations—including the higher potential toxicity of MC-LF—can be extrapolated to humans, as suggested by the findings of Fischer et al. (2010) from their experiments in primary human hepatocytes, these results not only explain the observed neurotoxicity in the Caruaru accident but also highlight the problems of using only one MC congener, MC-LR (Grosse et al. 2006; World Health Organization 1999), as the basis for MC-related human risk assessment.
